# Burnout in intensive care units - a consideration of the possible prevalence and frequency of new risk factors: a descriptive correlational multicentre study

**DOI:** 10.1186/1471-2253-13-38

**Published:** 2013-10-31

**Authors:** Carla Teixeira, Orquídea Ribeiro, António Manuel Fonseca, Ana Sofia Carvalho

**Affiliations:** 1 Department of Anaesthesia, Intensive Care and Emergency, Santo Antonio Hospital, Hospital Centre of Porto, Largo Prof. Abel Salazar, 4099-001, Porto, Portugal; 2 Department of Biostatistics and Medical Informatics, CINTESIS, Faculty of Medicine, University of Porto, Alameda Prof. Hernâni Monteiro, 4200-319, Porto, Portugal; 3 Centre for Studies in Human Development, Faculty of Education and Psychology, Catholic University of Portugal, Rua Diogo Botelho, 1327 4169-005, Porto, Portugal; 4 Institute of Bioethics, Catholic University of Portugal, Rua Diogo Botelho, 1327 4169-005, Porto, Portugal

**Keywords:** Burnout, Intensive Care, Risk factors

## Abstract

**Background:**

The provision of Intensive Care (IC) can lead to a health care provider’s physical, psychological and emotional exhaustion, which may develop into burnout. We notice the absence of specific studies regarding this syndrome in Portuguese Intensive Care Units (ICUs). Our main objective is to study the incidence and risk factors of burnout in Portuguese ICUs.

**Methods:**

A self-fulfilment questionnaire containing 3 items: (i) socio-demographic data of the study population; (ii) experiences in the workplace; (iii) Maslach Burnout Inventory (MBI) - was applied to evaluate the influence of distinct factors on the prevalence of burnout among physicians and nurses working in ICUs.

**Results:**

Three hundred professionals (82 physicians and 218 nurses) from ten ICUs were included in the study, out of a total of 445 who were eligible. There was a high rate of burnout among professionals working in Portuguese ICUs, with 31% having a high level of burnout. However, when burnout levels among nurses and physicians were compared, no significant difference was found. Using multivariate analysis, we identified gender as being a risk factor, where female status increases the risk of burnout. In addition, higher levels of burnout were associated with conflicts and ethical decision making regarding withdrawing treatments. Having a temporary work contract was also identified as a risk factor. Conversely, working for another service of the same health care institution acts as a protective factor.

**Conclusions:**

A high rate of burnout was identified among professionals working in Portuguese ICUs. This study highlights some new risk factors for burnout (ethical decision making, temporary work contracts), and also protective ones (maintaining activity in other settings outside the ICU) that were not previously reported. Preventive and interventive programmes to avoid and reduce burnout syndrome are of paramount importance in the future organization of ICUs and should take the above results into account.

## Background

### Context and purpose

Burnout was first described by Freudenberger in 1974. Delbrouck [1] characterizes this phenomenon as “[…] a state of fatigue or frustration motivated by dedication to a cause, a lifestyle or a relationship that did not meet expectations”. This syndrome has been studied primarily in the field of psychology by such authors as Maslach, Schaufeli and Leiter [2].

Burnout is specific to the work context, in contrast to depression, which tends to pervade every domain of a person’s life [2]. According to Maslach and Leiter [3], burnout results from inability to effectively manage chronic stress, which can be defined according to its multiple dimensions: emotional exhaustion (EE), depersonalization (DEP), and a reduction in personal and professional achievement (PPA).

There are high incidences of burnout in the helping professions due to the establishment of intense interpersonal relationships. Particularly in the context of health care, it is common for professionals to deal with situations which are complex and demanding, and which cause considerable stress. Burnout appears to be common among practi**s**ing physicians, with rates ranging from 25 to 60% [4].

Since many deaths in Intensive Care Units (ICUs) are preceded by a decision to withhold or withdraw life support, high-quality decision making [5] and end-of-life care are essential, as these can improve patient and family outcomes, as well as increasing the retention rate of clinicians working there. To make such a decision requires thorough training and good communication between the clinician and the family [6]. Coomber *et al*[7] reported that about a third of doctors in the UK seem to be under stress, while 10% reported symptoms of depression. Most attention has been focused on young doctors and their extended working hours [8]. However, there are also reports of stress among senior doctors [7]. Similarly, studies amongst Intensive Care (IC) nurses indicate that burnout is common, requiring urgent preventive measures [9].

The ICU (Intensive Care Unit) is characterized by a high level of work-associated stress [10], which is a factor known to increase the risk of burnout [11]. Studies in 1987 reported high levels of severe burnout in the case of IC nurses [12], and its association with decreased well-being among nursing teams [13] and costs related to absenteeism and a number of workplace changes [14], all of these having devastating consequences for the ICU and the health care system. According to Hinson and Spatz [15], in an era of constrained resources and nursing shortages, it is imperative to reduce staff turnover and increase the satisfaction of health care professionals. In 2008, Verdon *et al*[16] conducted some research into burnout among IC professionals. These authors found that a substantial proportion of nurses show symptoms of burnout and that organizational factors are predictive of levels of stress.

A study by Guntupalli, based on the MBI (Maslach Burnout Inventory), showed a high level of burnout among IC physicians, with the determinants associated with either patient care or lack of support felt by professionals [8].

As stated by Strack van Schijndel [17] conflicts can be useful and inevitable when people work together, but can also destroy an organization. A recent multicentre study by Poncet *et al*[18] of nurses working in ICUs showed that one third displayed severe symptoms of burnout syndrome. In this study it was found that conflicts with patients or between nurses and physicians contribute to burnout, while participation in research groups at ICUs protected against the onset of burnout.

Another recent publication by Embriaco *et al*[19] reveals high levels of burnout in ICUs. Half the intensivists studied reported a high level of burnout, which was associated with organizational factors. A study of Portuguese anaesthetists also showed high levels of burnout [20]. More recently, Michalsen and Hillert [21] reported an association between socio-demographic data and burnout. In their study older professionals experienced lower levels of burnout than younger ones, while female status was identified with higher levels of burnout.

However, we notice the lack of specific studies regarding this syndrome in Portuguese ICUs. In fact we have not identified any study that has previously evaluated the burnout syndrome in Portuguese ICUs involving both doctors and nurses. Therefore, we consider it expedient to conduct a study to assess burnout amongst IC physicians and nurses working in adult ICUs and aim to identify independent risk or protective burnout factors.

### Aims of the present study

Our main objective is to study the Portuguese situation regarding the incidence and risk factors of burnout in ICUs.

The specific aims are:

•To identify the levels of burnout of physicians and nurses working in ICUs comprising patients with differing needs.

•To identify factors that can lead to the development of burnout in physicians and nurses working in such a setting.

We proffer the following hypotheses: (*1*) there is a high rate of burnout among professionals working in Portuguese ICUs. (*2*) Burnout among intensivists and nurses is also associated with the following; (*a*) socio-demographic data; (*b*) the severity of patients’ illnesses; (*c*) organizational factors and work contexts such as workload and relationships with colleagues; and (*d*) ICU characteristics. Some of the results of these studies have been previously reported in the form of an abstract [22,23].

## Methods

### Design

Prospective observational transversal multicentre study.

### Selection of participants

All IC professionals (doctors and nurses) working in adult ICUs in state hospitals in the north of Portugal.

### Method of data collection

Each participating ICU received two types of documents. The first was to be filled in by the director of the unit and was designed to describe the IC setting: information about the ICU (teaching hospital or other, specific type of unit, number of beds), activity (number of admissions, duration of stay, Simplified Acute Physiology Score II [SAPS II] on admission, and mortality rate), number of physicians and nurses and their status, and patient-to-nurse ratio. Finally, ICU directors were asked to disclose whether there was a discussion group (a team forum for individuals to discuss various topics) and/or a psychologist in their unit.

The second document was a self-administered questionnaire for each physician and nurse working in the ICU. A covering letter outlining the purpose of the study, along with a three-part questionnaire, was also given to each participant. The covering letter stated that the purpose of the study was to develop a better understanding of the feelings of intensivists and nurses, and that the responses would be anonymous.

The questionnaire was divided into three parts.

**Part 1** Consisted of the MBI, a self-completion questionnaire developed by Maslach. The MBI is a 22-item questionnaire that has been shown to be reproducible and valid [2,24]. The inventory asks respondents to indicate on a seven point Lickert scale (which does not include the word “burnout”) the frequency with which they experience certain feelings related to their work. The MBI evaluates three domains of burnout. The EE subscale (nine items) assesses feelings of being emotionally overextended and exhausted by one’s work. The DEP subscale (five items) measures how unfeeling and impersonal is the response towards recipients of one’s service, care, or treatment. The PPA (eight items) assesses feelings of competence and successful achievement in one’s work with people. We defined cut-offs for burnout categories by considering the MBI Manual [24]. Answers to the MBI were used to classify the participants as having high, average or low levels of EE, DEP and PPA burnout dimensions. The following cut-offs were used to define low, average or high levels of each dimension of the MBI; EE: low, ≤ 14; average,15-24; high, ≥ 25; DEP: low, ≤ 3; average, 4–9; high, ≥ 10; PPA: low, ≥ 40 ; average, 33–39; high, ≤ 32 (inverse scale).

As the definition of burnout is controversial, we adopted the definition described in the literature: high levels of EE and DEP combined with low PPA. In order to define burnout risk, we state that the person presenting two of the three dimensions beyond the cut-off point is at high risk of burnout; the person with one of the three dimensions beyond the cut-off point is at average risk of burnout; the person showing average or low levels in the dimensions EE and DEP, and high or average levels in PPA, has a low risk of burnout. We considered a high level of burnout (high burnout) as the sum both of participants in burnout (B) and of those with a high risk of burnout (HR) [24,25].

**Part 2** Included basic demographic data (age, gender, marital status, number of children, religion, profession), professional activity, professional category, academic degrees, post-graduate training in IC, shift work, number of hours worked per week, time taken to reach work, years of professional experience, years of professional practice at the ICU, contractual situation, working in another department of the same or a different institution.

**Part 3** Consisted of recent experiences in the workplace. The ICUs are particularly demanding work contexts, either due to close dealings with end-of-life situations and the need for ethical decision-making, or because of the need to have multidisciplinary knowledge in order to achieve common goals. Therefore, we added another dimension to the questionnaire concerning one’s experiences in the work context based on a study by Embriaco *et al*[19]. Some questions concerned experiences during the previous week (number of patients under their care; night shift before survey fulfilment; whether off duty in the week/on the day before the survey; death of a patient; decision as to withholding/withdrawing treatments; conflicts with other professionals and/or with patients and families). Some more questions were asked about the existence of conflicts with other professionals. The respondents were also asked about their workload (mean number of working hours per week during the previous week, mean number of night shifts the previous week, time elapsed since their last week of holidays/last weekend or last day off duty).

### Ethical considerations

The study’s overall protocol was approved by the Institute of Bioethics of the Catholic University of Porto, Portugal. All ICU directors were contacted by letter. On the agreement form they had to indicate whether they consented to participate. For the implementation of the methodological tools, authorization of the relevant institutional bodies - the administration board, ethics committee and ICU directors - was required. The ethics committees that approved the study were those of the hospitals that were enrolled in the study, namely the ethics committees of the following (six) Hospitals: São João Hospital; Santo António Hospital-Hospital Centre of Porto; Pedro Hispano Hospital-Matosinhos; the Portuguese Oncology Institute of Porto; Hospital São Pedro, Vila Real-Hospital Centre of Trás os Montes and Alto Douro; and Viana de Castelo Hospital-Hospital Centre of Alto Minho.

The professionals who participated in the study were also asked for their informed consent. In addition, each instrument was preceded by a presentation sheet.

### Statistical analysis

In the descriptive analysis of the sample, summary statistics were applied as appropriate. The categorical variables were described by means of absolute frequencies (n) and relative (%) ones. Continuous variables were described using the median, 25th percentile and 75th percentile, since their distribution is asymmetric.A Chi-square independence test was used to examine the association between categorical variables. When the expected frequency in any cell of the contingency table analysis of the association of two categorical variables was less than 5, we used the Fisher’s exact test.

The Mann–Whitney and Kruskal-Wallis test respectively were used to test hypotheses comparing the distribution between 2 or more than 2 groups of continuous variables with asymmetric distribution. To ascertain risk factors associated with the existence of burnout, we determined odds ratios (OR) and confidence intervals (CI) at 95% by logistic regression. We performed a multivariate analysis to evaluate the independent relationship between burnout and some factors studied. To construct the multivariate logistic regression model independent variables were chosen for each outcome: those independent variables whose *p* < 0.05, and other variables (albeit not significant) described in the literature as predictors of primary importance. However, other significant variables which show a reduced number of cases were excluded, together with factors that might be related to each other and might influence the model (Additional file 1). Models of generalized estimating equations (GEE) were applied in order to adjust for the ICU cluster effect. Following the recommendations of Pepe and Fitzmaurice, we assume an exchangeable correlation matrix in the estimation of parameters [26,27]. We used a significance level of 0.05 for all hypothesis tests. The analysis was performed using the statistical analysis program SPSS® v.18.0.

## Results

A total of 10 out of the 13 (77%) ICU directors agreed to participate. Participating ICUs are situated in the same region of the country as selected ICUs, the only participating ICUs being those in the north of the country. The average number of ICU beds was 8 and the average length of stay in an ICU was 7 days, the mean SAPS II being 45; the ICU mortality average was 26%. 445 surveys, which included a return envelope addressed to the researchers, were distributed to ICUs that agreed to participate, 300 surveys being returned (a 67% response rate). No data was collected from the non-respondents (33%).

The sample comprised 300 professionals, 82 (27%) doctors and 218 (73%) nurses. The response rate was 78% amongst doctors and 64% amongst nurses. The average age was 32 years and 65% of the respondents were female.

**Figure 1:**
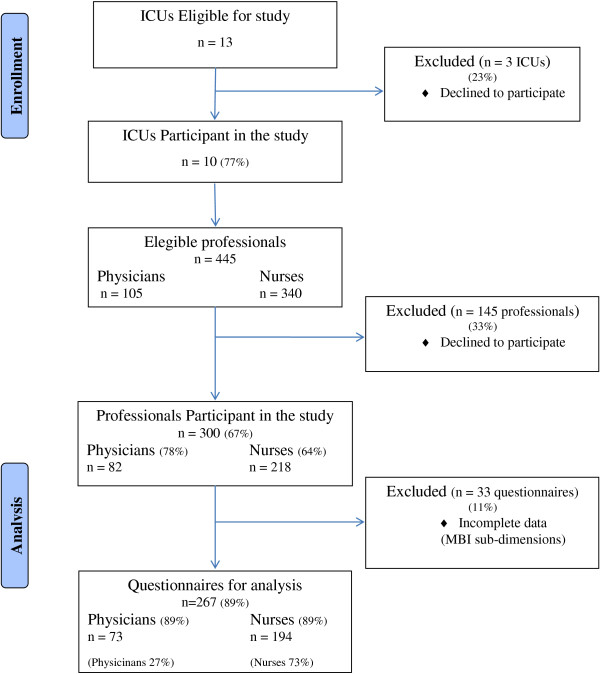
**Study design.** This flowchart describes the inclusion of professionals for eligible centers to arrive to the final study population (questionnaires considered for analysis). *ICU:* Intensive Care Unit.

From these 300 surveys, 33 (11%) questionnaires were excluded due to incomplete responses being received regarding the 3 MBI subdimensions. We identified professionals who had not responded to all items of burnout dimensions: 18 in the EE dimension (6%), 9 in the DEP dimension (3%) and 23 in the PPA dimension (8%). For burnout analysis we only considered questionnaires which were complete for the 3 subdimensions - 267 (89%) (Figure 1).

### Prevalence of burnout

Using the MBI, a high level of burnout was identified in 31% of participants (A high risk of burnout was identified in 22% of the respondents, while 9% were experiencing burnout). As for the remaining respondents, 39% of these presented a low risk of burnout while 30% indicated an average risk. DEP (a score of 10 or more is considered high) was observed in 27%, the mean level being 7 (±5). A high level of EE (a score of 25 or more is considered high) was present in 33% of the respondents, the mean level being 20 (±10). A low level of PPA (a score of 40 or more is considered low) was found in 23% of the 300 professionals who responded to the survey, the mean level being 34 (±8) (Table 1).

**Table 1:** Maslach Burnout Inventory - Three scale item distribution

	**n(%)**	**mean**	**(sd)**	**min**	**P25**	**med**	**P75**	**max**
Maslach burnout inventory								
**Emotional exhaustion**	282	20	(10)	0	12	19	27	50
Low (<=14)	106 (38)							
Average (15 a 24)	84 (30)							
High (> = 25)	92 (33)							
**Depersonalization**	291	7	(5)	0	3	5	10	22
Low (<=3)	104 (36)							
Average (4 a 9)	109 (37)							
High (> = 10)	78 (27)							
**Personal accomplishment**	277	34	(8)	8	28	34	39	48
Low (> = 40)	65 (23)							
Average (33 a 39)	97 (35)							
High (<=32)	115 (42)							
**Burnout**								
Low risk	104 (39)							
Average risk	80 (30)							
High risk (HR)	58 (22)							
In Burnout (B)	25 (9)							
**High level of Burnout (HB)**								
No	184 (69)							
Yes (HR + B)	83 (31)							

med-median; P-percentil; sd - standard deviation; min- minimum; max-maximum.

### Risk factors associated with burnout: univariate analysis

#### Personal and professional characteristics of the respondents

Female professionals reported higher levels of burnout than did males, yet in univariate analysis no statistically significant differences were found. Older professionals who had more years of professional experience presented a higher level of burnout. Respondents who reported a higher level of burnout were less likely to be married or partnered and often did not have children. Having a temporary work contract and not working for another service of the same institution were associated with a higher degree of burnout (Table 2). Workload (working hours per week, number of night shifts per month, lack of compensation for overtime, lack of respite since the last nonworking day or week) was not correlated to burnout.

**Table 2:** Univariate analysis of personal and professional burnout risk factors

	**ICU Professionals Total**	**High Burnout**		**OR**	**CI 95%**	***p***
		**No**	**Yes**			
	**(n = 267) (%)**	**(n = 184-69%)**	**(n = 83-31%)**			
**Gender**						
Female	173 (65)	116 (63)	57 (69)	1,000	-	0,373*
Male	94 (35)	68 (37)	26 (31)	0,778	0,448-1,351	
**Age**, med (P25-P75)	32 (27–38)	33 (28–40)	30 (27–36)	0,960	0,926-0,995	*0,011§*
**Marital Status**						
Single	122 (46)	74 (40)	48 (58)	1,000	-	*-*
Married	119 (45)	90 (49)	29 (35)	0,497	0,285-0,865	
Divorced	15 (6)	13 (7)	2 (2)	0,237	0,051-1,098	
Widower	1 (0)	0 (0)	1 (1)	-	-	
Other	10 (4)	7 (4)	3 (4)	0,661	0,163-2,680	
**With children**						
No	173 (65)	112 (61)	61 (74)	1,000	-	0,033*
Yes	93 (35)	72 (39)	21 (26)	0,536	0,301-0,954	
**Religion**						
No	68 (26)	46 (25)	22 (27)	1,000	-	0,747*
Yes	195 (74)	136 (75)	59 (73)	0,907	0,501-1,641	
**Profession**						
Physician	73 (27)	55 (30)	18 (22)	1,000	-	0,164*
Nurse	194 (73)	129 (70)	65 (78)	1,540	0,837-2,834	
**Academic qualifications**						
Bachelor / Graduation	250 (94)	175 (96)	75 (90)	1,000	-	*-*
Master	10 (4)	5 (3)	5 (6)	2,333	0,656-8,298	
PhD	5 (2)	2 (1)	3 (4)	3,500	0,573-21,376	
**Post-graduate course/training in Intensive Care**						
No	193 (75)	135 (76)	58 (73)	1,000	-	0,624*
Yes	63 (25)	42 (24)	21 (27)	1,164	0,634-2,137	
**Shift work**						
No	37 (14)	28 (15)	9 (11)	1,000	-	0,330**
Yes	229 (86)	155 (85)	74 (89)	1,485	0,667-3,307	
**Number of working hours (week)**						
35 hours	99 (37)	73 (40)	26 (31)	1,000	-	0,193*
40 hours	103 (39)	63 (34)	40 (48)	1,783	0,980-3,241	
42 hours	41 (15)	30 (16)	11 (13)	1,029	0,452-2,345	
Other	24 (9)	18 (10)	6 (7)	0,936	0,335-2,613	
**Distance between home and work** (Km), med (P25-P75)	7 (4–18)	7 (4–15)	10 (5–20)	1,008	0,995-1,022	*0,119§*
**Time taken to reach work** (minutes), med (P25-P75)	15 (10–30)	15 (10–30)	20 (10–30)	1,013	0,995-1,031	*0,274§*
**Years of professional experience**, med (P25-P75)	8 (4–14)	10 (4–15)	6 (4–13)	0,959	0,924-0,996	*0,048§*
**Years of professional practice in ICU**, med (P25-P75)	4 (2–9)	4 (2–10)	4 (1–7)	0,978	0,936-1,023	*0,244§*
**Contractual situation**, n (%)						
Effective staff member	114 (43)	89 (48)	25 (30)	1,000	-	0,002*
Individual contract of indeterminate period	109 (41)	62 (34)	47 (57)	2,699	1,506-4,837	
Fixed-term contract	17 (6)	15 (8)	2 (2)	0,475	0,102-2,216	
Without institutional link	4 (1)	4 (2)	0 (0)	-	-	
Other	23 (9)	14 (8)	9 (11)	2,289	0,887-5,904	
**Overschedule**						
In another setting from the same institution, n (%)						
No	212 (89)	139 (86)	73 (95)	1,000	-	0,040*
Yes	27 (11)	23 (14)	4 (5)	0,331	0,110-0,994	
In another health care institution, n (%)						
No	144 (58)	97 (58)	47 (57)	1,000	-	0,867*
Yes	104 (42)	69 (42)	35 (43)	1,047	0,613-1,788	

*OR - Univariate Odds Ratio; CI - Confidence Interval*. *High Burnout - High level of Burnout.*

med-median; P- Percentil; * Independence chi-square test ** Fisher exact test; § Mann–Whitney test.

In this study we found that the burnout levels of nurses in ICUs were not significantly different from those of physicians, although differences in burnout subdimensions were identified. Distributions of burnout subdimensions levels between physicians and nurses were the following: nurses exhibited higher levels of EE, while DEP and PPA were higher among physicians. Differences between nurses and physicians were identified in all three MBI components. However, these only reached statistical significance in the EE dimension (*p* = 0.019) (Table 3).

**Table 3:** Burnout dimensions among physicians and nurses

	**Total**		**Profession**				
			**Physician**		**Nurses**		
	med	(P25-P75)	med	(P25-P75)	med	(P25-P75)	*p§*
**Maslach Burnout Inventory:**							
- Emotional Exhaustion	19	(12-27)	17	(10-22)	20	(12-28)	*0,019*
- Depersonalization	5	(3-10)	6	(3-10)	5	(3-10)	0,229
- Personal and Professional Achievement	34	(28-39)	36	(31-41)	34	(27-38)	0,079

med-median; P-Percentile; § Mann–Whitney Test.

### Characteristics of the ICUs

The SAPS II score and mortality rate had an impact on burnout (Table 4). None of the ICUs included a formal discussion group or psychologist.

**Table 4:** Association between burnout and ICU characteristics

	**Total**	**High Burnout**		OR*	**CI 95%**	***p***
		**No**	**Yes**			
	**(n = 267)**	**(n = 184-69%)**	**(n = 83-31%)**			
**ICUs Characteristics**						
Number of beds, med (P25-P75)	8 (8–12)	8 (8–12)	10 (8–16)	1,089	0,970-1,222	*0,112§*
Annual number of admissions, med (P25-P75)	350 (300–413)	350 (300–413)	389 (300–413)	1,002	0,998-1,007	*0,020§*
Mean duration of hospitalization (days), med (P25-P75)	7 (6–10)	7 (6–12)	10 (6–10)	1,056	0,889-1,253	*0,307*
SAPS II score at admission, med (P25-P75)	45 (40–51)	45 (40–50)	50 (41–51)	1,040	0,989-1,093	*0,003§*
Mortality rate, med (P25-P75)	26 (16–32)	23 (14–26)	26 (19–32)	1,059	1,022-1,096	*0,001§*
Years of working in that service, med (P25-P75)	5 (4–7)	6 (4–7)	4 (4–7)	0,930	0,731-1,184	*0,034§*
Absenteeism n (%)						
No	231 (87)	163 (89)	68 (82)	1,000	-	0,140*
Yes	36 (13)	21 (11)	15 (18)	1,712	0,517-5,666	

High Burnout - High level of Burnout ; OR* - Univariate Odds Ratio; CI - Confidence Interval; P - Percentil; * Independence chi-square test; § Mann–Whitney test.

#### Experiences in the work context

The decision to withhold or withdraw treatment and to proceed to terminal sedation during the week preceding the survey had an impact on burnout. However, the death of one of the patients in a respondent’s care in the week before the survey was not a factor associated with a higher level of burnout. Conflicts were related to burnout. Indeed, higher levels of burnout were present in professionals who had had conflicts particularly with other colleagues or patients’ families (Table 5).

**Table 5:** Association between burnout and work experiences

	**ICU Professionals**	**High Burnout**		**OR**	**CI 95%**	***p***
	**Total**	**No**	**Yes**			
	**n = 267 (%)**	**n = 184 (69%)**	**n = 83 (31%)**			
**In the week before:**						
**Night shift**						
No	51 (20)	40 (23)	11 (14)	1,000	-	0,114*
Yes	200 (80)	134 (77)	66 (86)	1,791	0,864 -3,715	
**Additional shift**						
No	159 (65)	110 (65)	49 (66)	1,000	-	0,820*
Yes	85 (35)	60 (35)	25 (34)	0,935	0,526 -1,663	
**Off duty**						
No	83 (33)	61 (35)	22 (29)	1,000	-	0,328*
Yes	169 (67)	114 (65)	55 (71)	1,338	0,746-2,399	
**In holidays**						
No	222 (93)	150 (92)	72 (96)	1,000	-	0,255*
Yes	16 (7)	13 (8)	3 (4)	0,481	0,133-1,740	
**Death of one or more patients**						
No	113 (47)	84 (50)	29 (40)	1,000	-	0,155*
Yes	126 (53)	83 (50)	43 (60)	1,501	0,857-2,628	
**Conflicts, n (%)**						
No	193 (80)	141 (83)	52 (72)	1,000	-	0,046*
Yes	48 (20)	28 (17)	20 (28)	1,937	1,005-3,733	
**Conflicts with:**						
Colleagues n (%)						
No	222 (91)	157 (93)	65 (87)	1,000	-	0,082*
Yes	21 (9)	11 (7)	10 (13)	2,196	0,889-5,422	
Superiors n (%)						
No	229 (93)	162 (95)	67 (89)	1,000	-	0,124*
Yes	17 (7)	9 (5)	8 (11)	2,149	0,795-5,807	
Other professionals n (%)						
No	225 (91)	164 (96)	61 (81)	1,000	-	< 0,001*
Yes	21 (9)	7 (4)	14 (19)	5,377	2,072-13,955	
Patients n (%)						
No	242 (99)	170 (100)	72 (96)	1,000	-	0,028**
Yes	3 (1)	0 (0)	3 (4)	-	-	
Patient’s family n (%)						
No	229 (97)	158 (98)	71 (95)	1,000	-	0,213*
Yes	7 (3)	3 (2)	4 (5)	2,967	0,647-13,606	
**Ethical decisions:**						
Withhold treatments n (%)						
No	178 (73)	133	45	1,000	-	0,009*
Yes	66 (27)	38	28	2,178	1,203-3,943	
Withdraw treatments n (%)						
No	158 (65)	120	38	1,000	-	0,005*
Yes	85 (35)	50	35	2,211	1,256 - 3,891	
Terminal sedation n (%)						
No	180 (74)	134	46	1,000	-	0,010*
Yes	63 (26)	36	27	2,185	1,198 - 3,985	
Truth disclosure to patient’s n (%)						
No	172 (70)	116	56	1,000	-	0,256*
Yes	75 (30)	56	19	0,703	0,382 -1,294	
Truth disclosure to patient’s family						
No	106 (43)	77	29	1,000	-	0,439*
Yes	141 (57)	96	45	1,245	0,715-2,167	

High Burnout - High level of Burnout; OR - Univariate Odds Ratio; CI - Confidence Interval; P- Percentil; *Independence chi-square test; **Fisher exact test.

In addition, when we compared the 11% of partially completed questionnaires regarding the 3 MBI dimensions with the 89% which had been completed (Figure 1), in order to assess possible biases, no significant difference was found regarding recent experiences in the workplace. As regards socio-demographic and professional characteristics, no significant difference was found between professionals who supplied completed questionnaires and those who did not, except for age, years of professional experience, and years of professional practice in the ICU. Those who supplied partially completed questionnaires (who were excluded for the purpose of burnout analysis) were older (*p* = 0.039), had more years of professional experience (*p* = 0.027), and had had more practice in the ICU (*p* = 0.002) (Additional file 2).

As regards burnout analysis, there were no bias-associated factors due to incorrect completion of the questionnaire, as those professionals who were older and had had more years of professional experience (the majority of whom did not complete the questionnaire correctly), were also those with higher burnout levels. Furthermore, the number of years of professional experience in the ICU was found to be similar for professionals with both low and high burnout (Table 2).

### Risk factors associated with burnout: multivariate analysis

Using multivariate analysis, we identified gender as being a risk factor, where female status together with conflicts and withdrawing treatments increase the risk of burnout. Conversely, working for another service of the same health care institution acts as a protective factor. Nevertheless, even though age and some ICU characteristics are related to burnout in univariate analysis, the same does not apply in multivariate analysis (Table 6).

**Table 6:** Multivariate analyses of burnout risk factors

	**Total (n = 267)**	**High Burnout**		OR**	**CI95%**	**p**
		**No**	**Yes**			
		**n = 184 (69%)**	**n = 83 (31%)**			
**Gender**, n (%)						
Female	173 (65)	116 (63)	57 (69)	1,000	-	
Male	94 (35)	68 (37)	26 (31)	0,530	0,349-0,806	0,003
**Overschedule**						
**In another setting in the same institution**, n (%)						
No	212 (89)	139 (86)	73 (95)	1,000	-	
Yes	27 (11)	23 (14)	4 (5)	0,167	0,034-0,805	0,026
**Conflicts e**, n (%)						
No	193 (80)	141 (83)	52 (72)	1,000	-	
Yes	48 (20)	28 (17)	20 (28)	2,323	1,347-4,007	0,014
**Ethical decisions**, n (%)						
**Withdraw treatments**						
No	158 (65)	120 (71)	38 (52)	1,000	-	
Yes	85 (35)	50 (29)	35 (48)	2,123	1,266-3,560	0,004
**Withhold treatments**						
No	178 (73)	133 (78)	45 (62)	1,000	-	
Yes	66 (27)	38 (22)	28 (38)	1,711	0,937-3,124	0,080
**Age**, med (P25-P75)	32 (27–38)	33 (28–40)	30 (27–36)	0,966	0,872-1,069	0,504
**Marital status**, n (%)						
Single	122 (46)	74 (40)	48 (58)	1,000	-	
Married	119 (45)	90 (49)	29 (35)	0,914	0,387-2,158	0,837
Divorced/Widower/Other	26 (10)	20 (11)	6 (7)	0,630	0,123-3,237	0,580
**With children**, n (%)						
No	173 (65)	112 (61)	61 (74)	1,000	-	
Yes	93 (35)	72 (39)	21 (26)	1,576	0,534-4,651	0,410
**Profession**, n (%)						
Physician	73 (27)	55 (30)	18 (22)	1,000	-	
Nurse	194 (73)	129 (70)	65 (78)	0,736	0,195-2,771	0,650
**Number working hours (week)**, n (%)						
35 hours	99 (37)	73 (40)	26 (31)	1,000	-	
40 hours	103 (39)	63 (34)	40 (48)	0,733	0,257-2,089	0,561
42 hours	41 (15)	30 (16)	11 (13)	1,340	0,341-5,269	0,675
Other	24 (9)	18 (10)	6 (7)	0,994	0,278-3,553	0,993
**Contractual situation**, n (%)						
Effective staff member	114 (43)	89 (48)	25 (30)	1,000	-	
Contract of indeterminate period	109 (41)	62 (34)	47 (57)	2,380	0,669-8,462	0,180
Fixed-term contract/Without institutional link/Other	44 (16)	33 (18)	11 (13)	0,770	0,148-4,016	0,756
**Death of a patient**, n (%)						
No	113 (47)	84 (50)	29 (40)	1,000	-	
Yes	126 (53)	83 (50)	43 (60)	0,787	0,325-1,910	0,597
**Terminal sedation**						
No	180 (74)	134 (79)	46 (63)	1,000	-	
Yes	63 (26)	36 (21)	27 (37)	0,456	0,193-1,078	0,074
**SAPS II**, med (P25-P75)	45 (40–51)	45 (40–50)	50 (41–51)	0,974	0,822-1,156	0,767
**Mortality**, med (P25-P75)	26 (16–32)	23 (14–26)	26 (19–32)	1,108	0,957-1,282	0,171

*High Burnout - High level of Burnout*; OR**- Multivariate Odds Ratio; CI -Confidence Interval; med-median; P-Percentil.

## Discussion

Physicians and nurses working in Portuguese ICUs who participated in the study present a high level of burnout (31%). In fact, burnout appears to be common among practising ICU professionals [4,28], with rates ranging from 25 to 60%.

In relation to the MBI subdimensions we found that the DEP level was similar to that in a study by Guntupalli and Fromm [8], whereas PPA and EE rates were found to be slightly lower.

When comparing the Portuguese ICU characteristics with those of Embriaco’s study, we found that the mean duration of hospitalization was similar. However, the number of beds per unit was smaller in the Portuguese units. Patients admitted to Portuguese ICUs were more seriously ill (as shown by a higher SAPS II) and the mortality rate was also higher. In spite of this, when the results of our study were compared with those of Embriaco *et al*[19], a lower percentage of professionals were found to have burnout. A high level of burnout was identified in 31% of the respondents (46.5% in the Embriaco study). While the number of professionals with a low level of PPA was similar (42% vs 39% in the Embriaco study), those displaying DEP were lower in number (27% vs 37% in the Embriaco study). Finally, the major difference between the two studies was the high level of EE found in 33% of the ICU respondents in the current study (compared with 19% in the Embriaco study). Some of the differences may be due to the fact that in our study nurses and physicians were enrolled simultaneously, while in Embriaco’s study only physicians were enrolled. When considering our data we also found higher DEP levels in physicians and higher EE in nurses. Actually, in spite of the fact that nurses’ burnout levels were not significantly different from those of physicians, we did identify some differences in burnout subdimensions. The nurses exhibited higher levels of EE, while DEP and PPA were higher among physicians. Indeed, differences between nurses and physicians were identified in all three components of burnout. However, this only gained statistical significance in the EE dimension (*p* = 0.019). Patient care orientations assumed by nurses and physicians have long been recognized to be inherently different [29]. Critical care physicians and nurses have discrepant attitudes to the teamwork experience. Overall, physicians appear more satisfied with physician-nurse collaboration than nurses. Data presented by Thomas *et al*[30] suggest this different global rating of teamwork may be attributable to several specific issues; nurses reported that in their dealings with physicians it is difficult to speak up, disagreements are not appropriately resolved, more input into decision-making is needed, and nurse input is not well received. The nursing approach emphasizes a more personal affiliation with patients, which often puts individual nurses in direct conflict with physicians, whose responsibilities may oblige them to assume a more emotionally neutral or technical perspective toward patients [29]. High stress levels occur as a result of conflicts which nurses’ psychosocial and emotional resources are unable to cope with [31]. This could explain some of our results as nurses showed higher EE and physicians higher DEP.

According to current findings, ageing and length of years of professional practice were identified as risk factors for burnout. However, multivariate analysis did not confirm these results. Other findings were presented by Michalsen and Hiller [21], according to whom older professionals displayed lower levels of burnout.

We found that being female increases the risk of burnout. Our results after multivariate analysis, such as those presented in Embriaco’s study, found that gender was the only personal characteristic that is independently associated with burnout (Table 2). This is not in line with the studies reviewed by Thomas [32], none of which demonstrated a higher risk or differential effect of burnout for women, nor does it correspond with the studies of Tironi *et al,* in which the greatest prevalence rates of burnout were observed among younger male physicians [33]. Nevertheless, in a recent study conducted in Germany, female status was also identified as presenting higher levels of burnout [21].

For Maslach *et al*[2], burnout is a response to overload. Generally, workload is related to the EE dimension. For Gopal *et al*, reducing hours may be the first step in decreasing resident burnout [34]. However, in the present study, overall workload alone is not associated with higher burnout (Table 2). For Thomas, restricting work hours alone is not associated with a reduction in burnout [32]. In a study of Australian anaesthesiologists, improving work organization (presence of skilled assistance in the operating theatre) was the most important factor leading to reduced burnout [35].

We also found higher risk of developing burnout in professionals with short term contracts when compared with those with long-term contracts. It was previously reported [36,37] that compared to permanent employees, fixed term employees reported lower levels of workload, job security, and job satisfaction. It was also stated that a higher psychological morbidity could be found among temporary workers compared with permanent employees [38].

We found a reduced risk of burnout in professionals who also work in another department of the institution. Those who work in ICUs and also elsewhere in the same institution exhibit a lower risk of burnout, which is confirmed as being an independent variable. Maintaining activity in another department of the institution is, as far as we know, a newly identified burnout protective factor. This works in favor of not creating IC as a primary speciality and must be taken into account when considering future organization and training in IC. Furthermore, our results are in accordance with the editorial of Van Acken *et al*. Transforming Intensive Care Medicine (ICM) into a primary speciality would disqualify professionals from working in another specialism, whereas the ‘particular qualification’ concept allows them to return to their ‘mother disciplines’ or to rotate there for some time [39]. As ICM is extremely demanding, both physically and mentally, one would expect severe problems to occur with physicians who will have to leave ICM after some years due to ‘burnout’ [40]. Thus a ‘particular qualification’ in an area of expertise in addition to a primary speciality qualification is required not only to provide high-quality patient care, but also for the wellbeing of the professional (in order to reduce burnout risk). Our results support ICM becoming a supraspeciality involving a multidisciplinary approach in order to facilitate high-level care for the critically ill patient.

SAPS II score, mortality rate (Table 4), and decision to withhold or withdraw treatment (Table 5), were associated with a higher level of burnout. In contrast to Embriaco’s study [19] (where patient characteristics were not independently associated with a higher level of burnout) and in accordance with the study of Baldwin [41], we found a correlation between the mortality rate among patients and the degree of burnout. We also found higher levels of burnout in professionals who need to suspend or limit treatment or provide terminal sedation. Nevertheless, in our study, when a multivariate analysis was conducted, only withdrawing treatment appeared to contribute to higher burnout levels (Table 6). These results were also found by Embriaco *et al*[19], according to whom withdrawing treatments is a burnout risk factor. As previously stated by Savulesco [42], there is some ambiguity over the distinctions in both theory and practice between easing and hastening death, and this discontinuation of the treatment might be interpreted as equivalent to accepting a hastened dying process [43]. A point of interest to note is that the death of one patient is not in itself related to burnout, but the manner of dying is. This emphasizes the importance of implementing ethical deliberations at end of life in IC settings.

We found that the risk of burnout increases when conflict and serious disagreement arise with someone in the week prior to completing the questionnaire. This is even greater when there is a conflict with other professionals. Conflicts negatively impact on patient safety, patient-family-centred care and team welfare and cohesion. They generate staff burnout and increase healthcare costs [44]. For Stehle [45], many of the stressors identified concerned working relationships between nurses and doctors. For young Swiss physicians (residents and chief residents), the most important job expectations were good relationships with colleagues [46]. On the other hand, effective team work and good leadership, management, support and supervision appear to be protective factors that need further enhancement [47].

Concerning burnout and ICU characteristics, we also found, in addition to SAPS II and the mortality rate, that the number of patients admitted per year to the ICU is associated with burnout (the higher the number, the greater the risk). In addition, professional dissatisfaction, represented by reduced years of work in ICUs, was also identified as a risk factor (Table 4). Nonetheless, none of these factors has been confirmed by multivariable analysis.

In the present study, those professionals with the highest rate of burnout had a higher level of absenteeism. Although that was not statistically significant, there were a greater number of professionals with high risk of burnout who were still working. In the study by Embriaco *et al*[19], about 50% of the intensivists exhibiting a high level of burnout wish to leave their jobs. However, for people who stay in their jobs, burnout leads to lower productivity and effectiveness at work. Consequently, it is associated with decreased professional satisfaction and reduced commitment to the job or the organization. Previous investigations in a wide variety of settings have shown that burnout may affect performance [48] and quality of medical services [49]. In a study of medical residents, a high DEP level was associated with self-reported suboptimal patient care practices [50]. Burnout has been associated with various forms of job withdrawal–absenteeism, intention to leave the job, and turnover.

One of the main approaches to improving the quality of care of patients hospitalized in the ICU is to ensure that the caregivers are in “tune” with their patients. Some authors suggested that burnout can be reduced by an intensive communication strategy that brings about quicker end-of-life decision making in the ICU. Quenot *et al*[51] state that personal accomplishment is increased through measures that help to give meaning to work, particularly through intensive communication strategies adopted by healthcare teams.

The strengths of this study are that it has a prospective multi-sited design, which includes a variety of different ICUs, and a considerable number of participants (as evidenced by the high response rate), who constitute professionals (nurses and physicians) working in ICUs. It also includes the use of standardized measures (MBI).

Notwithstanding these strengths, there are some limitations that need to be considered. Firstly, despite data from ten different ICUs, the survey was not conducted countrywide, so the extent to which generalizations can be made relating to the entire country is limited. These findings encourage us to plan a study involving those units. However, taking the country as a whole, there are no major cultural differences, participating ICUs being similar to non-participating ones. Moreover, despite the fact that differences may exist between the characteristics of Portuguese ICUs and ICUs in other countries (some of these being previously reported), as well as between the attributes and ICU experiences of Portuguese professionals when compared with those of other countries, we consider that the results of this study could also be relevant for non-Portuguese ICUs. Secondly, although the rate of response to the questionnaire was high and representative, the rate of non-responders still amounts to 33%. We cannot exclude the possibility that these collaborators did not complete the questionnaire because they had a high degree of burnout and were unable to participate, were on sick leave due to work causes or had already left the ICU because they were burned out. Thirdly, we did not ask the professionals directly about their intention to resign from their positions, nor about the quality of their relationships, but only about conflicts. Fourthly, we stress the limitations of a purely quantitative study in this especially sensitive area. The classic scientific procedure may be excessively reductive when applied to “human subjects”. We propose further exploration of qualitatively designed research, such as that of Chahraoui in relation to the subjective and emotional experience of health care professionals in ICUs [52].

## Conclusions

Caring for acutely ill patients may lead to burnout syndrome, and its occurrence was associated with socio-demographic data: being married and having children actually reduced the risk of occurrence. Using multivariate analysis, we identified gender as being a risk factor, where female status increased the risk of burnout.

Burnout in intensivists and nurses was associated with organizational factors and the work context, such as workload and experiences in the workplace. An important new association between burnout and work organization was identified. Working in ICUs and also elsewhere in the same institution was associated with a reduced risk of burnout, which led us to think that maintaining professional activities in other areas besides the ICU is a protective factor in terms of burnout. As far as we know, this is a new and very important finding, which in our opinion must be taken into account not only with regard to the future training of ICU professionals, but also in relation to the organization of ICUs.

Professional groups were each affected in different ways by burnout subdimensions, thus fostering the need for different interventions amongst professional teams in order to decrease burnout. These measures need to focus on decreasing EE and increasing PPA amongst nurses, and on decreasing DEP amongst doctors. Further research is also necessary to explore and understand burnout and related risk factors amongst professional groups.

Conflicts with colleagues were associated with a higher level of burnout. Ways of promoting good inter-professional relationships (such as team meetings, interdisciplinary case discussions and supervisions) and of investigating as a team with clear responsibilities could lead to a healthy workplace and could serve as useful strategies for fostering engagement and preventing burnout. In fact, changing working conditions and managing professional conflicts are essential for dealing with this syndrome.

The occurrence of burnout was influenced by the ethical decision-making related to end of life care. One of the most important decisions health care teams need to make in ICUs is related to withdrawing life support. We found an association between this decision and burnout, which reinforces the notion that professionals have an enormous responsibility with regard to patient survival and that they must be supported and trained in order to proceed to ethical deliberation regarding end of life decision making.

This study highlights some new burnout-associated factors, both increasing the risk of burnout (ethical decision making regarding end of life issues, temporary work contracts), and also reducing it (maintaining activity in other settings outside the ICU). In our opinion preventive and interventive programmes to avoid and reduce burnout syndrome are of paramount importance in the future organization of ICUs and should take the above results into account.

### Key messages

•Caring for acutely ill patients may lead to burnout syndrome, the occurrence of which was influenced by ethical decision-making related to end of life care. When conducting a multivariate analysis, withdrawing treatment was associated with higher burnout levels.

•Working in ICUs and also elsewhere in the same institution was associated with a lower risk of burnout, which led us to think that maintaining professional activities in other areas besides the ICU may reduce the risk of burnout.

•This study highlights the need for preventive and interventive programmes in order to avoid and reduce burnout syndrome in ICUs, focusing on the team, on organizational areas, on dealing with conflicts and on ethical deliberation, particularly regarding end of life issues.

•These results might help ICU staff and hospital/unit managers to gain awareness of the existence of burnout and thus to take corrective action in order to reduce its occurrence.

## Abbreviations

GEE: Generalized estimating equations; IC: Intensive Care; ICM: Intensive Care Medicine; ICU: Intensive Care Unit; ICUs: Intensive Care Units; MBI: Maslach Burnout Inventory; SAPS II: Simplified Acute Physiology Score II; OR: Odds ratios; CI: Confidence intervals; EE: Emotional exhaustion; DEP: Depersonalization; PPA: Personal and Professional Accomplishment; HB: High level of Burnout; HR: High risk of burnout; B: In burnout.

## Competing interests

All authors declare that they have no competing interests.

## Authors’ contributions

CT and AMF conceived and designed the study. CT drafted the manuscript and collected data. OR and CT undertook the statistical analysis. CT, AMF and ASC contributed to the review and revisions of the manuscript. CT wrote the final manuscript. All authors read and approved the final manuscript.

## Pre-publication history

The pre-publication history for this paper can be accessed here:

http://www.biomedcentral.com/1471-2253/13/38/prepub

## Supplementary Material

Additional file 1 Multivariate analyses of burnout risk factors – without including Profession. Click here for file

Additional file 2 Comparison of the partially completed questionnaires regarding the 3 MBI dimensions with those that were fully completed. Click here for file

## References

[B1] Delbrouck M Síndrome de Exaustão (Burnout) 2006 Lisboa: Climepsi Editores

[B2] Maslach C Schaufeli WB Leiter MP Job burnout Annu Rev Psychol 2001 52 397 422 10.1146/annurev.psych.52.1.397 11148311

[B3] Maslach C Leiter MP The truth about Burnout. How Organizations cause personal stress and what to do about it 1997 San Francisco, CA: Jossey-Bass

[B4] Grassi L Magnani K Psychiatric morbidity and burnout in the medical profession: an Italian study of general practitioners and hospital physicians Psychother Psychosom 2000 69 329 334 10.1159/000012416 11070446

[B5] Lundgren-Laine H Kontio E Perttila J Korvenranta H Forsstrom J Salantera S Managing the daily intensive care activities - an observation study concerning ad hoc decision-making of charge nurses and intensivists Crit Care 2011 15 R188 10.1186/cc10341 21824420 PMC3387631

[B6] Curtis JR Vincent JL Ethics and end-of-life care for adults in the intensive care unit Lancet 2010 375 1347 1353 10.1016/S0140-6736(10)60064-5 20934213

[B7] Coomber S Todd C Park G Baxter P Firth-Cozens J Shore S Stress in UK intensive care unit doctors Br J Anaesth 2002 89 873 881 10.1093/bja/aef273 12453932

[B8] Guntupalli KK Fromm RE Jr Burnout in the internist–intensivist Intensive Care Med 1996 22 625 630 10.1007/BF01709737 8844225

[B9] Chen SM McMurray A Burnout in intensive care nurses J Nurs Res 2001 9 152 164 10.1097/01.JNR.0000347573.45553.e0 11779088

[B10] Donchin Y Seagull FJ The hostile environment of the intensive care unit Curr Opin Crit Care 2002 8 316 320 10.1097/00075198-200208000-00008 12386492

[B11] Bakker AB Le Blanc PM Schaufeli WB Burnout contagion among intensive care nurses J Adv Nurs 2005 51 3 276 287 10.1111/j.1365-2648.2005.03494.x 16033595

[B12] Soupios MA Lawry K Stress on personnel working in a critical care unit Psychiatr Med 1987 5 187 198 3328879

[B13] Aiken LH Clarke SP Sloane DM Sochalski J Silber JH Hospital nurse staffing and patient mortality, nurse burnout, and job dissatisfaction JAMA 2002 288 1987 1993 10.1001/jama.288.16.1987 12387650

[B14] Ackerman AD Retention of critical care staff Crit Care Med 1993 21 Suppl 9 394 395 10.1097/00003246-199309001-00063 8365251

[B15] Hinson TD Spatz DL Improving nurse retention in a large tertiary acute-care hospital J Nurs Adm 2011 41 Suppl 3 103 108 21336037 10.1097/NNA.0b013e31820c7242

[B16] Verdon M Merlani P Perneger T Ricou B Burnout in a surgical ICU team Intensive Care Med 2008 34 152 156 10.1007/s00134-007-0907-5 17943271

[B17] van Schijndel RJM S Burchardi H Bench-to-bedside review: Leadership and conflict management in the intensive care unit Crit Care 2007 11 R234 10.1186/cc6108 PMC2246194 18086322

[B18] Poncet MC Toullic P Papazian L Kentish-Barnes N Timsit JF Pochard F Chevret S Schlemmer B Azoulay E Burnout syndrome in critical care nursing staff Am J Respir Crit Care Med 2007 175 698 704 10.1164/rccm.200606-806OC 17110646

[B19] Embriaco N Azoulay E Barrau K Kentish N Pochard F Loundou A Papazian L High level of burnout in intensivists Am J Respir Crit Care Med 2007 175 686 692 10.1164/rccm.200608-1184OC 17234905

[B20] Morais A Maia P Azevedo A Amaral C Tavares J Stress and Burnout among Portuguese Anesthesiologists Eur J Anaesthesiology 2006 23 433 439 10.1017/S0265021505001882 16469205

[B21] Michalsen A Hillert A Burnout in anesthesia and intensive care medicine. Part 1.Clarification and critical evaluation of the term Anaesthesist 2011 60 23 30 10.1007/s00101-009-1659-0 20094692

[B22] Teixeira C Fonseca A Carvalho AS Pereira SM Burnout in Intensive Care Units [abstract] Intensive Care Med 2010 36 S155

[B23] Teixeira C Pereira SM Ribeiro O Fonseca A Carvalho AS Burnout in ICUs in Portugal: is there? Are there differences between doctors and nurses? [abstract] Crit Care 2011 15 S172

[B24] Maslach C Jackson SE Leiter MP The Maslach Burnout Inventory manual 1996 3 Palo Alto, CA: Consulting Psychologiss Press

[B25] Ramirez AJ Graham J Richards MA Burnout and psychiatric disorder among cancer clinicians Br J Cancer 1995 71 1263 1269 10.1038/bjc.1995.244 7540037 PMC2033827

[B26] Pepe MS Anderson GL A cautionary note on inference for marginal regression models with longitudinal data and general correlated response data Communs Statist. Simuln Computn 1994 23 939 951 10.1080/03610919408813210

[B27] Fitzmaurice GM Laird NM Ware JH Applied Longitudinal Analysis 2004 Hoboken, NJ: Wiley-Interscience

[B28] Gallery ME Whitley TW Klonis LK Anzinger RK Revicki DA A study of occupational stress and depression among emergency physicians Ann Emerg Med 1992 21 58 64 10.1016/S0196-0644(05)82238-3 1539889

[B29] Corser WD The contemporary nurse-physician relationship: insights from scholars outside the two professions Nurs Outlook 2000 48 263 268 10.1067/mno.2000.109154 11135138

[B30] Thomas EJ Sexton JB Helmreich RL Discrepant attitudes about teamwork among critical care nurses and physicians Crit Care Med 2003 31 956 959 10.1097/01.CCM.0000056183.89175.76 12627011

[B31] Cronqvist A Theorell T Burns T Lützén K Dissonant imperatives in nursing: a conceptualization of stress in intensive care in Sweden Intensive Crit Care Nurs 2001 17 228 236 10.1054/iccn.2000.1588 11868731

[B32] Thomas NK Resident burnout JAMA 2004 292 2880 2889 10.1001/jama.292.23.2880 15598920

[B33] Tironi MO Nascimento Sobrinho CL Barros DS Reis EJ Marques Filho ES Almeida A Bitencourt A Feitosa AI Neves FS Mota IC França J Borges LG Lordão MB Trindade MV Teles MS Almeida MB Souza YG Professional burnout syndrom among intensive care physicians in Salvador, Brazil Rev Assoc Med Bras 2009 56 656 662 20191219 10.1590/s0104-42302009000600009

[B34] Gopal R Glasheen JJ Miyoshi TJ Prochazka AV Burnout and internal medicine resident work-hour restrictions Arch Intern Med 2005 165 2595 2600 10.1001/archinte.165.22.2595 16344416

[B35] Kluger MT Townend K Laidlaw T Job satisfaction, stress and burnout in Australian specialist anaesthetists Anaesthesia 2003 58 339 345 10.1046/j.1365-2044.2003.03085.x 12648115

[B36] Virtanen M Kivimäki M Elovainio M Vahtera J Ferrie JE From insecure to secure employment: changes in work, health, health related behaviours, and sickness absence Occup Environ Med 2003 60 948 953 10.1136/oem.60.12.948 14634187 PMC1740437

[B37] Virtanen M Kivimäki M Joensuu M Virtanen P Elovainio M Vahtera J Temporary employment and health: a review Int J Epidemiol 2005 34 610 622 15737968 10.1093/ije/dyi024

[B38] Jennings BM Rockville (MD) Work stress and burnout among nurses: role of the work environment and working conditions Patient Safety and Quality: An Evidence-Based Handbook for Nurses 2008 26 US: Agency for Healthcare Research and Quality 21328768

[B39] Van Aken H Olsen JM Pelosi P Intensive care medicine: a multidisciplinary approach! Eur J Anaesthesiol 2011 28 313 315 10.1097/EJA.0b013e328345a441 21487261

[B40] Raggio B Malacarne P Burnout in intensive care unit Minerva Anestesiol 2007 73 195 200 17468736

[B41] Baldwin PJ Dodd M Wrate RM Young doctors’ health–II. Health and health behaviour Soc Sci Med 1997 45 41 44 10.1016/S0277-9536(96)00307-3 9203269

[B42] Savulesco J End-of-life decisions Medicine 2005 33 Suppl 2 11 15

[B43] Rydvall A Lynöe N Withholding and withdrawing life-sustaining treatment: a comparative study of the ethical reasoning of physicians and the general public Crit Care 2008 12 R13 10.1186/cc6786 18279501 PMC2374603

[B44] Fassier T Azoulay E Conflicts and communication gaps in the intensive care unit Curr Opin Crit Care 2010 16 Suppl 6 654 665 20930623 10.1097/MCC.0b013e32834044f0

[B45] Stehle JL Critical care nursing stress: the findings revisited Nurs Res 1981 30 182 186 7015292

[B46] Biaggi P Peter S Ulich E Stressors, emotional exhaustion and aversion to patients in residents and chief residents: what can be done? SwissMed Wkly 2003 133 339 346 10.4414/smw.2003.10134 12923685

[B47] Onyett S Revisiting job satisfaction and burnout in community mental health teams J Ment Health 2011 20 198 209 10.3109/09638237.2011.556170 21406021

[B48] Weisman CS Teitelbaum MA Physician gender and the physician–patient relationship: recent evidence and relevant questions Soc Sci Med 1985 20 1119 1127 10.1016/0277-9536(85)90189-3 3895448

[B49] McCue JD The effects of stress on physicians and their medical practice N Engl J Med 1982 306 458 463 10.1056/NEJM198202253060805 7057844

[B50] Shanafelt TD Bradley KA Wipf JE Back AL Burnout and self-reported patient care in an internal medicine residency program Ann Intern Med 2002 136 358 367 10.7326/0003-4819-136-5-200203050-00008 11874308

[B51] Quenot JP Rigaud JP Prin S Suffering among careers working in critical care can be reduced by an intensive communication strategy on end-of-life practices Intensive Care Med 2012 38 55 61 10.1007/s00134-011-2413-z 22127481 PMC3678991

[B52] Chahraoui K Bioy A Cras E Gilles F Laurent A Valache B Quenot JP Psychological experience of health care professionals in intensive care unit: a qualitative and exploratory study Ann Fr Anesth Reanim 2011 30 4 342 348 10.1016/j.annfar.2011.01.020 21411265

