# Supplementary material for: Burnout in intensive care units - a consideration of the possible prevalence and frequency of new risk factors: a descriptive correlational multicentre study
Source: BMC Anesthesiol. 2013 Oct 31;13:38. doi: 10.1186/1471-2253-13-38 (PMC3826848; doi:10.1186/1471-2253-13-38)
Supplement: Additional file 1 — Multivariate analyses of burnout risk factors – without including Profession. [file 1471-2253-13-38-S1.pdf]

Electronic File 1 - Multivariate analyses of burnout risk factors – without including Profession

	Total (n=267)	High Burnout No (n=184-69%)	High Burnout Yes (n=83–31%)	OR**	CI 95%	p
**GENDER**, n (%)						
Female	173(65)	116(63)	57(69)	1,000	-	
Male	94(35)	68(37)	26(31)	**0,534**	**0,347-0,820**	**0,004**
**OVERSCHEDULE**						
**In another setting in the same institution**, n (%)						
No	212(89)	139(86)	73(95)	1,000	-	
Yes	27(11)	23(14)	4(5)	**0,173**	**0,040-0,749**	**0,019**
**CONFLICTS e**, n (%)						
No	193(80)	141(83)	52(72)	1,000	-	
Yes	48(20)	28(17)	20(28)	**2,279**	**1,308-3,969**	**0,004**
**ETHICAL DECISIONS**, n (%)						
**WITHDRAW TREATMENTS**						
No	158(65)	120(71)	38(52)	1,000	-	
Yes	85(35)	50(29)	35(48)	**2,126**	**1,262-3,582**	**0,005**
**WITHHOLD TREATMENTS**						
No	178(73)	133(78)	45(62)	1,000	-	
Yes	66(27)	38(22)	28(38)	1,777	0,969-3,259	**0,063**
**AGE**, med (P25-P75)	32(27-38)	33(28-40)	30(27-36)	0,976	0,909-1,048	0,497
**MARITAL STATUS**, n (%)						
Single	122(46)	74(40)	48(58)	1,000	-	
Married	119(45)	90(49)	29(35)	0,907	0,401-2,051	0,814
Divorced/Widower/Other	26(10)	20(11)	6(7)	0,619	0,124-3,099	0,560
**WITH CHILDREN**, n (%)						
No	173(65)	112(61)	61(74)	1,000	-	
Yes	93(35)	72(39)	21(26)	1,645	0,586-4,612	0,344
**NUMBER WORKING HOURS (week)**, n (%)						
35 hours	99(37)	73(40)	26(31)	1,000	-	
40 hours	103(39)	63(34)	40(48)	0,769	0,268-2,202	0,624
42 hours	41(15)	30(16)	11(13)	1,605	0,293-8,777	0,585
Other	24(9)	18(10)	6(7)	1,188	0,385-3,668	0,764
**CONTRACTUAL SITUATION**, n (%)						
Effective staff member	114(43)	89(48)	25(30)	1,000	-	
Contract of indeterminate period	109(41)	62(34)	47(57)	2,625	0,971-7,099	0,057
Fixed-term contract / Without institutional link/ Other	44(16)	33(18)	11(13)	0,914	0,358-2,331	0,851
**DEATH OF A PATIENT**, n (%)						
No	113(47)	84(50)	29(40)	1,000	-	
Yes	126(53)	83(50)	43(60)	0,792	0,330-1,903	0,602
**TERMINAL SEDATION**						
No	180(74)	134(79)	46(63)	1,000	-	
Yes	63(26)	36(21)	27(37)	0,459	0,191-1,104	0,082
**SAPS II**, med (P25-P75)	45(40-51)	45(40-50)	50(41-51)	0,977	0,822-1,160	0,790
**MORTALITY**, med (P25-P75)	26(16-32)	23(14-26)	26(19-32)	1,107	0,955-1,282	0,178

High Burnout - High level of Burnout; OR**- *Multivariate Odds Ratio; CI -Confidence Interval; med-median; P-Percentil;*
