# Supplementary material for: Burnout in intensive care units - a consideration of the possible prevalence and frequency of new risk factors: a descriptive correlational multicentre study
Source: BMC Anesthesiol. 2013 Oct 31;13:38. doi: 10.1186/1471-2253-13-38 (PMC3826848; doi:10.1186/1471-2253-13-38)
Supplement: Additional file 2 — Comparison of the partially completed questionnaires regarding the 3 MBI dimensions with those that were fully completed. [file 1471-2253-13-38-S2.pdf]

Electronic File 2 - Comparison of the partially completed questionnaires regarding the 3 MBI dimensions with those that were fully completed

	ICU Professionals		Questionnaires				
	**Total (n=300)**		**Incomplete (n=33-11%)**		**Complete (n=267-89%)**		*p*
**1. Gender**							
Female	195	(65)	22	(67)	173	(65)	0,832*
Male	105	(35)	11	(33)	94	(35)	
**2. Age**, med (P25-P75)	32	(28-39)	36	(29-45)	32	(27-38)	***0,039§***
**3. Marital Status**							
Single	133	(44)	11	(33)	122	(46)	-
Married	139	(46)	20	(61)	119	(45)	
Divorced	15	(5)	0	(0)	15	(6)	
Widower	2	(1)	1	(3)	1	(0)	
Other	11	(4)	1	(3)	10	(4)	
**4. With children** No	192	(64)	19	(58)	173	(65)	0,399*
Yes	107	(36)	14	(42)	93	(35)	
**5. Religion** No	77	(26)	9	(27)	68	(26)	0,861*
Yes	219	(74)	24	(73)	195	(74)	
**6. Profession**							
Physician	82	(27)	9	(27)	73	(27)	0,993*
Nurse	218	(73)	24	(73)	194	(73)	
**7. Academic qualifications**							
Bachelor / Graduation	280	(94)	30	(91)	250	(94)	0,433**
Master / PhD	18	(6)	3	(9)	15	(6)	
**8. Post-graduate training in Intensive Care**							
No	215	(75)	22	(69)	193	(75)	0,416*
Yes	73	(25)	10	(31)	63	(25)	
**9. Shift work**							
No	45	(15)	8	(24)	37	(14)	0,125**
Yes	254	(85)	25	(76)	229	(86)	
**10. Number of working hours (week)**							
35 hours	114	(38)	15	(45)	99	(37)	0,142*
40 hours	110	(37)	7	(21)	103	(39)	
42 hours	46	(15)	5	(15)	41	(15)	
Other	30	(10)	6	(18)	24	(9)	
**11.Distance between home and work** (Km), med (P25-P75)	7	(4-15)	8	(6-15)	7	(4-18)	0,519§
**12. Time taken to reach work** (minutes), med (P25-P75)	15	(10-30)	15	(10-25)	15	(10-30)	0,650§
**13. Years of professional experience**, med (P25-P75)	8	(4-15)	12	(7-20)	8	(4-14)	***0,027§***
**14. Years of professional practice in ICU**, med (P25-P75)	4	(2-10)	8	(4-13)	4	(2-9)	***0,002***§
**15. Contractual situation**, n (%)							
Effective staff member	134	(45)	20	(61)	114	(43)	-
Individual contract of indeterminate period	118	(39)	9	(27)	109	(41)	
Fixed-term contract	18	(6)	1	(3)	17	(6)	
Without institutional link	4	(1)	0	(0)	4	(1)	
Other	26	(9)	3	(9)	23	(9)	
**16. Overschedule :**							
**16.1.** In another setting from the same institution , n (%)							
No	241	(90)	29	(97)	212	(89)	0,336**
Yes	28	(10)	1	(3)	27	(11)	
**16.2.** In another health care institution , n (%)							
No	158	(56)	14	(44)	144	(58)	0,124*
Yes	122	(44)	18	(56)	104	(42)	

*med-median; P- Percentil; * Independence chi-square test ** Fisher exact test; § Mann-Whitney test*
.
